# Myocardial Revascularization in 2025: A Clinical Perspective on the Evolution of Technologies, Strategic Decision-Making, and Future Horizons

**DOI:** 10.31083/RCM45516

**Published:** 2026-03-23

**Authors:** Vaibhav Sharma, Kriti Ahuja

**Affiliations:** ^1^Department of Internal Medicine, Medstar Washington Hospital Center, Washington, DC 20010, USA; ^2^Department of Internal Medicine, Kasturba Medical College, 575001 Mangalore, India

**Keywords:** myocardial revascularization, coronary artery disease, percutaneous coronary intervention, coronary artery bypass, drug-eluting stents, chronic total occlusion, robotic surgical procedures, artificial intelligence, precision medicine, hybrid procedures

## Abstract

Coronary artery disease remains the leading cause of death worldwide, causing the field of myocardial revascularization to evolve rapidly. This review synthesizes current evidence and emerging trends, providing clinicians with practical guidelines to support decision-making in practice. Current drug-eluting stents have attained excellent safety profiles, with restenosis rates below 3%. Percutaneous treatment of complex lesions is now routinely feasible, with success rates of 90–95% in experienced institutions. Surgical revascularization remains the standard of care for complex multivessel disease, and total arterial grafting provides a strong long-term survival advantage. Nonetheless, emerging technologies, such as artificial intelligence (AI)-guided interventions, robotic interventions, and precision medicine strategies, have the potential to overcome current limitations and extend advanced therapies to high-risk patients. The optimal revascularization plan increasingly depends on integrating anatomical complexity, physiological significance, patient-specific features, and institution-specific expertise. Heart team-based decision-making is now a necessity, particularly in difficult cases where hybrid strategies might offer particular advantages. Over the coming decade, the extensive use of AI-assisted procedural planning, the broader adoption of minimally invasive treatments, and the establishment of prescription-based personalized medicine protocols are likely to be observed. Success will depend on addressing current challenges, including health disparities, delayed complications, and increasing heterogeneity in the patient population.

## 1. Introduction: The Contemporary Challenge

Consider this clinical scenario: A 68-year-old patient with diabetes, triple-vessel coronary artery disease, moderate left ventricular dysfunction, and chronic kidney disease; twenty years ago, the treatment decision was straightforward—surgical revascularization was the only option. Today, this patient could receive a drug-eluting stent, robotic-assisted minimally invasive coronary surgery, hybrid revascularization, or traditional bypass surgery, all with a distinct risk–benefit profile.

**Fig. 1.**
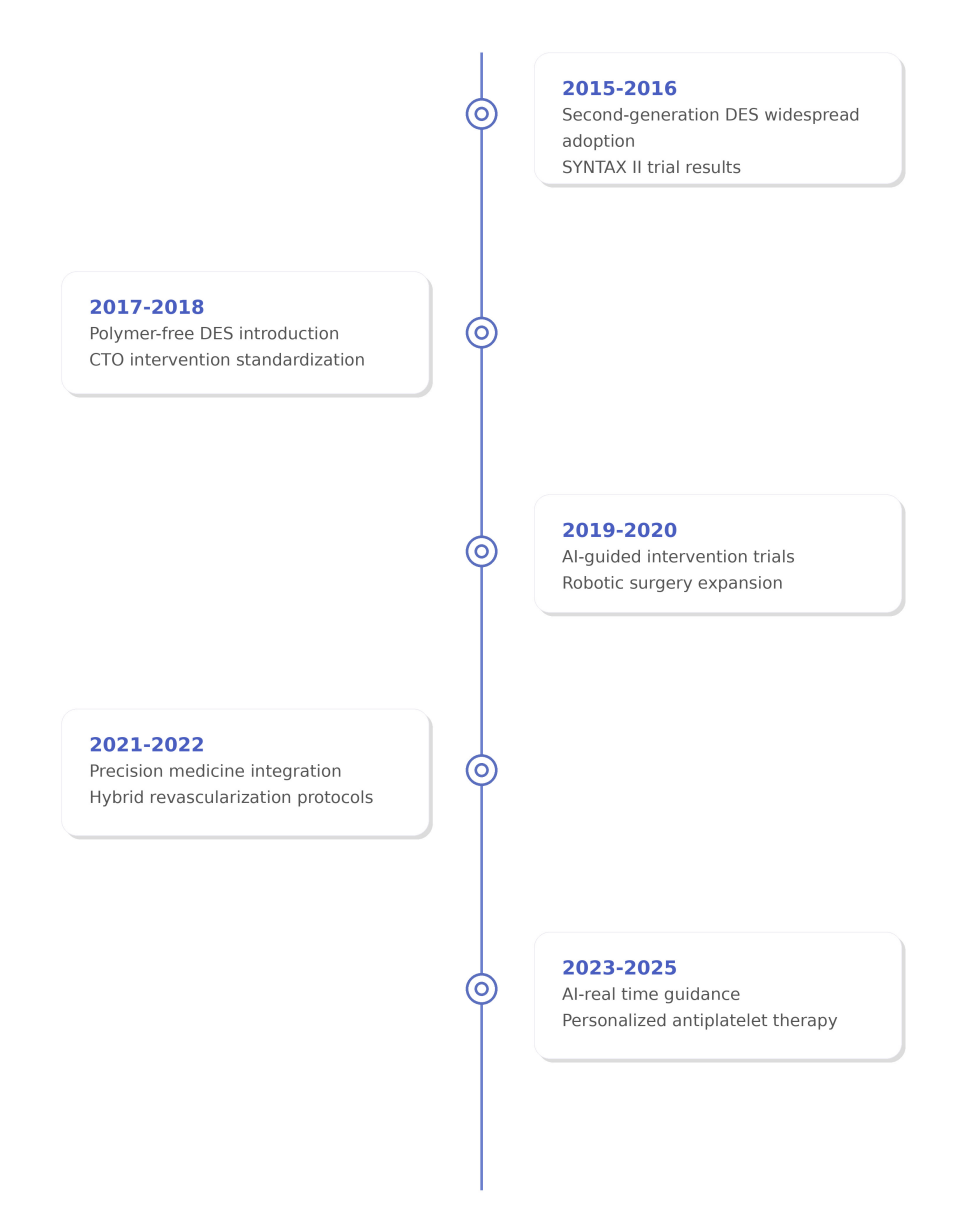
**Timeline of major technological advances in myocardial revascularization (2015–2025) of the rapid evolution of therapeutic options and clinical evidence**. Key: DES, drug-eluting stent; CTO, chronic total occlusion; AI, artificial intelligence. Figure created with Biorender.

This transition reflects the evolution of myocardial revascularization over the past decade. While coronary artery disease remains responsible for 8.9 million deaths globally each year, with a disproportionate impact on low- and middle-income nations [1], our therapeutic arsenal has expanded dramatically. However, these advances have also introduced new challenges: increasingly complex patient populations, ongoing healthcare disparities, and the need to incorporate rapidly developing technologies into evidence-based practice (Fig. 1 highlights major recent technological advancements that could form the foundation for future advances).

### 1.1 The Current Paradigm Shift

Contemporary revascularization practice faces three key challenges that distinguish this practice from previous options. First, patient complexity has increased dramatically: patients today are older, have multiple comorbidities and frailty, and present atypically, making classical risk–benefit analysis difficult [2]. Second, technological advancement has outpaced evidence generation, forcing physicians to extrapolate from limited data when applying new devices and technologies in real-world patients. Third, healthcare delivery systems must balance new technology with equal access, as geographic and socioeconomic disparities persist amid overall improvements in outcomes [3].

### 1.2 Why This Review Matters Now

This clinical narrative synthesis addresses these challenges by pragmatically filtering the current evidence, prioritizing decision-making over exhaustive reporting of all available data. Unlike systematic reviews that primarily aggregate trial outcomes, our approach emphasizes the intersection of new technology with timeless principles and acts as a guide for clinicians navigating an increasingly complex therapeutic landscape. We believe that effective modern-day revascularization demands a paradigm shift from procedure-oriented to patient-oriented decision-making, one that includes anatomical and physiological considerations, as well as patient values, institutional capacity, and long-term care coordination.

## 2. Methods

### 2.1 Search Strategy and Evidence Selection

We conducted a comprehensive literature search in the PubMed, EMBASE, and the Cochrane Library databases from January 2019 to December 2024. Search terms included combinations of “myocardial revascularization”, “percutaneous coronary intervention”, “coronary artery bypass grafting”, “drug-eluting stents”, “artificial intelligence”, “robotic surgery”, and “precision medicine”. Additional targeted searches addressed specific clinical scenarios, including chronic total occlusions, left main disease, and hybrid revascularization.

### 2.2 Inclusion and Exclusion Criteria

**Inclusion criteria:** Randomized controlled trials, large observational studies (n >1000), systematic reviews and meta-analyses, major society guidelines, and landmark registry analyses published in peer-reviewed journals were reviewed. Preference was given to studies reporting clinical endpoint data and long-term follow-up of more than two years. The focused timeframe of 2019–2024 was intentionally selected to capture contemporary practice patterns, current-generation devices, and emerging technologies (e.g., AI, robotics, precision medicine) that define modern revascularization. Earlier landmark trials are appropriately referenced through recent guidelines and comparative studies that build on these foundational works.

**Exclusion criteria:** Case reports, small single-center studies (n <100), studies without clinical endpoints, conference abstracts without full-text publication, and publications not available in English were excluded.

### 2.3 Quality Assessment and Evidence Synthesis

The quality of evidence was graded using standardized instruments: the GRADE approach (Grading of Recommendations Assessment, Development and Evaluation) for randomized trials, the Newcastle–Ottawa scale for observational studies, and AMSTAR-2 (A Measurement Tool to Assess Systematic Reviews, version 2) for systematic reviews. Synthesis placed the highest priority on Level 1 evidence from randomized controlled trials, supplemented by observational data to enhance clinical context and real-world applicability. Study differences were addressed by close methodological congruence and by focusing on study populations, endpoints, and follow-up periods.

## 3. Current Revascularization Strategies: Evidence and Clinical Application

### 3.1 Percutaneous Coronary Intervention: Expanding Boundaries

#### 3.1.1 The Drug-Eluting Stent Evolution

Modern drug-eluting stents represent one of the most significant technological advances in cardiology. Contemporary devices have stent restenosis rates below 3%, and polymer-free designs achieve even lower rates while avoiding the chronic inflammation associated with permanent synthetic polymers [4, 5]. Long-term follow-up from major trials now confirms stent thrombosis rates below 0.5%, thereby revolutionizing the risk–benefit balance compared with surgical revascularization [6].

New stent platforms have extended the limits of percutaneous coronary intervention (PCI) into areas once considered surgical. In patients at high risk of bleeding or those who require short dual antiplatelet therapy, polymer-free drug-eluting stents are especially useful. However, second-generation devices remain the gold standard for most clinical applications [6].

New issues must also be addressed in current practice, including genetic predispositions to procedural adversity. Stress-induced ventricular arrhythmias can unmask underlying *RyR2* mutations, which carry significant prognostic implications for procedural risk stratification [7]. Even incidental findings, such as endocardial calcification on preprocedural imaging, must be thoroughly assessed because they can affect both procedural approach and long-term outcomes [8].

#### 3.1.2 Complex Lesion Interventions: Achieving Consistent Success

3.1.2.1 Chronic Total Occlusions: From Specialized to Standard Practice

Contemporary chronic total occlusion (CTO) interventions have evolved significantly, with success rates of 90–95% and severe complication rates below 2% in high-volume centers [9]. This improvement is due to the systematic application of evidence-based methods rather than operator skill alone.

Present-day CTO interventions rely on advanced techniques and emphasize organized approaches, including dual angiography with extensive lesion assessment, microcatheter use for accurate guidewire manipulation, and supportive crossing techniques such as antegrade wiring, dissection–reentry, and retrograde techniques (Table 1; Ref. [9, 10, 11, 12]).

**Table 1.** **Contemporary CTO success factors**.

Factor	Impact on success	Clinical consideration
Dual angiography	+15–20% success rate	Essential for retrograde assessment
Microcatheter use	+10–15% success rate	Enables precise guidewire control
Hybrid approach	+20–25% success rate	Requires operator expertise in all techniques
Intravascular imaging	+5–10% success rate	Guides optimal stent sizing and deployment
Operator experience	Critical determinant	Annual volumes >75 cases for optimal outcomes

Adapted from Brilakis *et al*. [9, 10], Carlino *et al*. [11], and Goel *et al*. [12]. CTO, chronic total occlusion.

The modern strategy involves a structured patient assessment starting with verification of an ischemic presentation and imaging to evaluate viable myocardium in the bed of the occluded artery [13]. Assessment of anatomical complexity with the Japan chronic total occlusion (J-CTO) score yields useful prognostic data, with scores ≥2 predicting increased procedural complexity [9]. Institutional preparedness should include the availability of all crossing and bailout techniques needed to manage potential complications [14, 15].

3.1.2.2 Bifurcation Lesions: Evidence-Based Simplification

Bifurcation lesions, which account for approximately 20% of all PCI procedures, provide a classic example of how evidence-based simplification can improve outcomes. Encouragement from the European Bifurcation Club for a provisional stenting strategy has reduced procedural complexity while achieving excellent outcomes in most lesions [16]. Two-stent strategies are now reserved for complicated bifurcations with large side branches (>2.75 mm in diameter, >70% stenosis, large myocardial territory), as routine use has been associated with increased complications without improved outcomes.

3.1.2.3 Left Main Disease: The Evolving Heart Team Decision

Left main PCI poses the greatest challenge to traditional surgical dogma, not only technically but also in terms of appropriate patient selection, as reflected by the SYNTAX score analysis, life expectancy, surgical risk, and patient preference [17, 18]. This involves careful consideration of anatomical complexity based on the revised SYNTAX score criteria: scores below 22 generally favor percutaneous treatment, scores of 23–32 require a case-by-case decision, and scores above 32 typically support surgical superiority.

Risk stratification for surgery using established scores, such as the Society of Thoracic Surgeons (STS) score, identifies anatomically suitable patients in whom the risk of surgery (>4%) would favor percutaneous treatment [19]. Factors such as older age (>80 years) and major comorbidities significantly influence treatment choice, as these factors favor minimal access treatment even in the presence of anatomical complexity [20, 21].

### 3.2 Surgical Revascularization: Evolving Excellence

#### 3.2.1 The Arterial Grafting Imperative

As the indications for PCI have expanded, surgical revascularization has focused on achieving optimal long-term outcomes through total arterial grafting. The existing evidence demonstrates beyond doubt the superiority of arterial conduits over venous conduits, and total arterial revascularization is associated with significant 10-year reductions in cardiac mortality and improved event-free survival [22, 23].

#### 3.2.2 Long-Term Patency Rates by Conduit Type

∙ Left internal mammary artery to left anterior descending: >95% at 10 years.

∙ Radial artery: >90% at 10 years.

∙ Saphenous vein: ~60% at 10 years.

∙ Right internal mammary artery: >85% at 10 years.

The problem is not in identifying arterial dominance but in safely applying this concept across all patient populations. Registry analyses show that even for single arterial grafting (left internal mammary artery to left anterior descending), this procedure is associated with significant mortality benefits compared with venous grafts alone [24].

#### 3.2.3 On-Pump Versus Off-Pump: Evidence-Based Selection

The off-pump versus on-pump controversy has been transformed from an ideological to an evidence-based, patient-focused choice. Current evidence indicates that although off-pump coronary artery bypass grafting has short-term benefits in selected subsets (e.g., low ejection fraction, older individuals), the concern is the completeness of revascularization rather than the method *per se* [25, 26].

Patient factors such as advanced age (>80 years) and severe comorbidities, including end-stage renal disease, are likely to predispose toward off-pump methods because of decreased systemic inflammatory response [27]. However, the skill of the surgeon is crucial, and an annual off-pump case volume of more than 100 cases is associated with outcomes comparable to those of on-pump surgery [28, 29, 30].

#### 3.2.4 High-Risk Surgical Populations: Expanding Boundaries Safely

Contemporary cardiac surgery is increasingly being performed in high-risk groups of patients who were once considered inoperable. Severe left ventricular ejection fraction dysfunction (≤30%) is best managed by minimizing postoperative complications with prophylactic mechanical support devices [31, 32]. Nutritional status has also emerged as a significant, yet underappreciated risk factor; preoperative nutritional optimization is a modifiable variable that can, in many cases, more than compensate for benefits in high-risk patients [33].

### 3.3 Comparative Effectiveness: The Heart Team Approach

#### 3.3.1 When Surgery Remains Superior

Despite advances in PCI, coronary artery bypass grafting remains unequivocally superior in certain patient subsets. Current real-world evidence indicates that, in patients with severe multivessel disease, especially those with diabetes, surgical revascularization is superior for long-term survival and freedom from major adverse cardiac and cerebrovascular events (Table 2; Ref. [17, 34, 35]).

**Table 2.** **Contemporary CABG vs. PCI decision framework**.

Clinical scenario	Preferred strategy	Key considerations
Left main + complex multivessel (SYNTAX >32)	CABG	Clear mortality benefit, especially with diabetes
Isolated left main, low complexity (SYNTAX <22)	PCI	Particularly if high surgical risk
Three-vessel disease with diabetes	CABG	FREEDOM trial 10-year data support surgery
Two-vessel disease, including proximal LAD	Heart team decision	Consider functional assessment, patient factors
Single-vessel disease	PCI	Unless surgical indication exists

Based on Park *et al*. [17], Tam *et al*. [34], and contemporary SYNTAX score analyses [35]. Key: CABG, coronary artery bypass graft; LAD, left anterior descending; PCI, percutaneous coronary intervention.

**Fig. 2.**
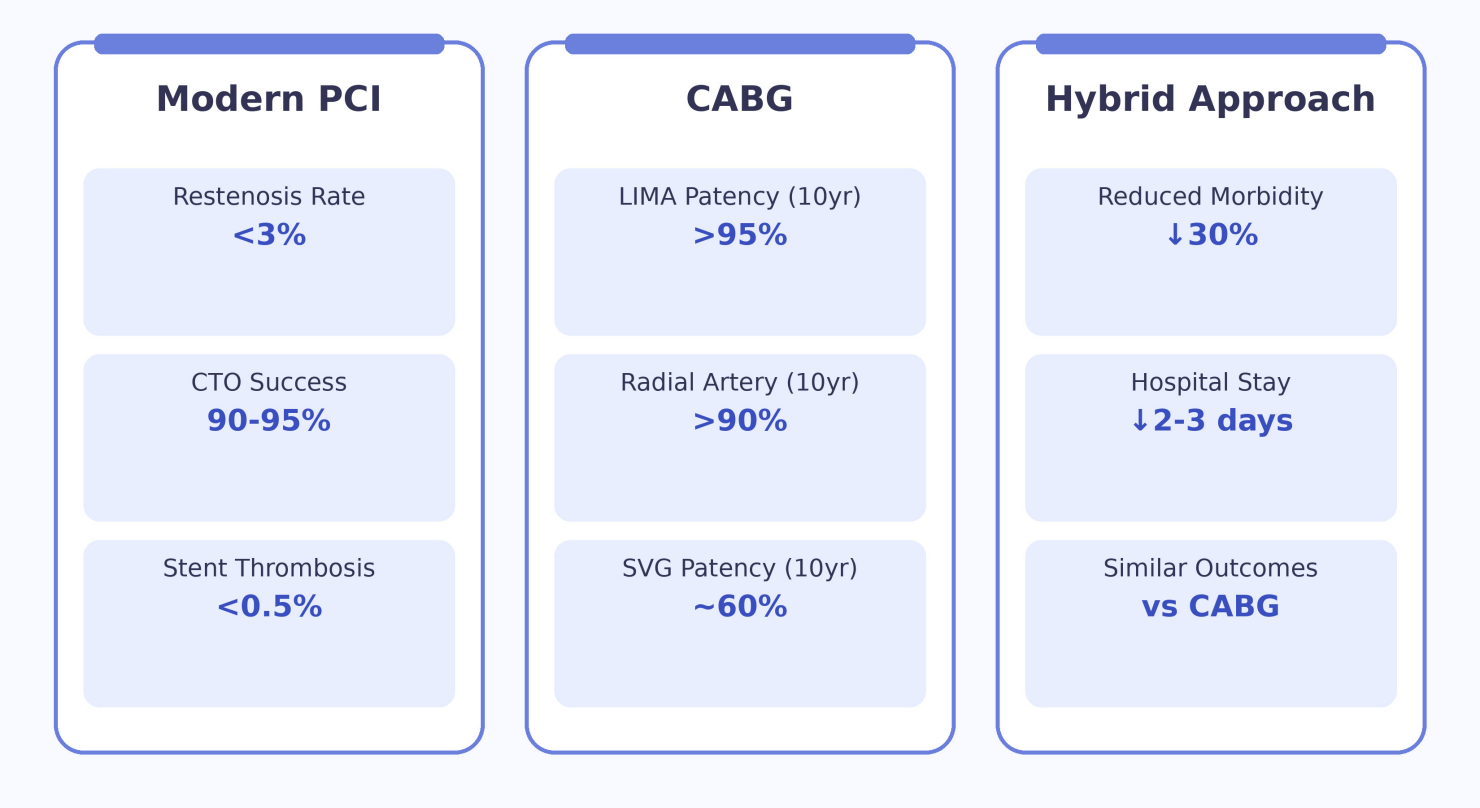
**Contemporary clinical outcomes across revascularization strategies [4, 5, 6, 23, 24, 36, 37]**. LIMA, Left Internal Mammary Artery; SVG, Saphenous Vein Graft. Figure created by Biorender.

This model acknowledges the sophistication of modern decision-making, acknowledging that patient choice, institutional practices, and individual risk profiles must all be incorporated into treatment decisions (Fig. 2; Ref. [4, 5, 6, 23, 24, 36, 37]).

#### 3.3.2 Quality Metrics and Outcome Optimization

Modern quality improvement requires holistic measurements that capture procedural success, patient-focused outcomes, and sustainable effectiveness. Implementing high-quality programs is associated with improved clinical outcomes, fewer readmissions, and more efficient resource utilization [38].

## 4. Emerging Technologies and Strategic Innovations

### 4.1 Artificial Intelligence: Transforming Clinical Practice

#### 4.1.1 Artificial Intelligence-Guided Procedural Planning and Execution

Artificial intelligence (AI) technologies for cardiac interventions have the highest potential to transform the field, comparable to the impact of drug-eluting stents. Machine learning algorithms outperform traditional risk scores in predicting mortality and complications, and AI-assisted procedural planning enables real-time optimization of device selection and deployment strategy [39].

#### 4.1.2 Current AI Applications

∙ Risk stratification: Superior prediction of procedural outcomes compared to traditional scores.

∙ Lesion assessment: Automated fractional flow reserve calculation from angiography.

∙ Device selection: Optimal stent sizing and positioning guidance.

∙ Outcome prediction: Enhanced long-term prognosis assessment.

#### 4.1.3 Near-Term Developments (2025–2027)

∙ Real-time procedural guidance systems with automated complication detection.

∙ Personalized dual antiplatelet therapy duration recommendations.

∙ Integration with electronic health records for comprehensive decision support.

∙ Predictive analytics for complex coronary anatomy identification [40].

### 4.2 Robotic Surgery: Precision and Enhanced Outcomes

#### Current Capabilities and Clinical Results

Robotic cardiac surgery has progressed from an experimental technique to standard practice at large centers, demonstrating reproducible benefits, including shorter operative times, reduced blood loss, lower rates of conversion to open surgery, and fewer postoperative complications [41]. These benefits translate into shorter hospital stays and potential cost savings, despite the higher initial equipment costs. Modern robotic cardiac surgery offers several evidence-based benefits: the minimally invasive surgery reduces surgical trauma, high-definition three-dimensional (3D) visualization enhances precision, and improved ergonomics support better surgeon performance [42, 43, 44, 45].

While there are benefits, there are also restrictions to highlight: a steep learning curve of at least 75–100 cases is required to achieve maximum proficiency, high costs related to equipment and maintenance costs, rigid case selection that typically excludes reoperations and emergency cases, and the requirement for comprehensive backup facilities to enable immediate conversion to open surgery if required [46, 47, 48, 49, 50, 51].

### 4.3 Precision Medicine: Personalized Revascularization Strategies

#### 4.3.1 Biomarker-Guided Therapy

Current biomarker data allow tracking of patients requiring complex coronary revascularization and facilitate optimization of procedural planning [40]. Inflammatory biomarkers such as interleukin-6 and resistin correlate with coronary disease progression and post-procedural complications, enabling targeted interventions and maximized medical therapy [52].

Biomarker-directed therapy encompasses the entire revascularization spectrum: pre-procedural risk stratification and strategy selection, prediction of procedural complications and intra-procedural adjustments, post-procedural monitoring with tailored recovery protocols, and long-term residual risk assessment for augmented prevention strategies [53, 54, 55, 56, 57].

#### 4.3.2 Pharmacogenomics in Antiplatelet Therapy

Platelet function and genetic polymorphism testing assist in tailoring the choice of antiplatelet treatment. Genetic loss-of-function defects in *CYP2C19* alleles identify patients with reduced clopidogrel response, and platelet function testing guides therapy optimization [57]. Current evidence favors switching from clopidogrel to ticagrelor in STEMI patients with high platelet inhibition, with considerably improved one-year outcomes without an increased bleeding risk [58].

### 4.4 Hybrid Revascularization: Integrating Optimal Strategies

#### 4.4.1 Strategic Integration of PCI and CABG

Hybrid coronary revascularization combines the long-term durability of arterial grafting with the lower morbidity of PCI. Modern practice often consists of left internal mammary artery-to-left anterior descending artery grafting using minimally invasive methods, followed by PCI of the remaining vessels [36].

#### 4.4.2 Optimal Hybrid Candidates

∙ Anatomical: Complex left anterior descending disease with moderate non-left anterior descending disease.

∙ Clinical: High surgical risk requiring durable left anterior descending revascularization.

∙ Technical: Suitable anatomy for a minimally invasive surgical approach.

∙ Institutional: Expertise in both minimally invasive surgery and complex PCI.

Current evidence supports hybrid revascularization as an equally effective alternative, with comparable short- and long-term results, but reduced perioperative morbidity relative to conventional CABG [37].

## 5. Clinical Decision-Making in Contemporary Practice

### 5.1 Comprehensive Patient Assessment

#### 5.1.1 Integrating Multiple Assessment Modalities

Contemporary patient selection necessitates the systematic integration of multiple assessment tools in addition to traditional angiographic evaluation [11]. Anatomical assessment involves high-resolution coronary angiography with quantitative analysis and complexity grading using a modified SYNTAX criteria [35]. Intravascular imaging with optical coherence tomography or intravascular ultrasound provides detailed plaque characterization to guide optimal device selection [12].

Physiological assessment with fractional flow reserve or instantaneous wave-free ratio measurements guarantees revascularization of hemodynamically relevant stenoses [59]. Stress testing and viability imaging help determine the optimal strategy in patients with reduced ejection fractions [60, 61].

#### 5.1.2 Coronary Computed Tomography Angiography: Expanding the Diagnostic Arsenal

Coronary CTA has emerged as a transformative, non-invasive imaging modality that provides a comprehensive assessment of coronary anatomy, plaque characteristics, and perivascular inflammation. Modern platforms enable precise anatomical visualization with excellent correlation to invasive angiography, facilitating patient screening and procedural planning, particularly for complex lesions such as chronic total occlusions, bifurcation disease, and left main stenoses [47, 48].

Computed tomography (CT)-derived fractional flow reserve (CT-FFR) enables non-invasive hemodynamic assessment of stenosis severity with strong diagnostic accuracy compared with invasive FFR, identifying ischemia-producing lesions that require revascularization while helping to avoid unnecessary interventions [47]. Quantitative plaque analysis identifies high-risk features, including positive remodeling, low-attenuation plaque, and spotty calcification, thereby informing both the urgency of revascularization and the intensification of medical therapy. Perivascular fat attenuation index quantification detects coronary inflammation with prognostic implications [48].

For surgical planning, coronary CTA provides comprehensive visualization of coronary anatomy and potential graft conduits, enabling preoperative assessment of the suitability of the internal mammary artery, radial artery, and saphenous vein for conduit selection [48]. Current guidelines support coronary CTA as a first-line diagnostic test in patients with stable chest pain and an intermediate pre-test probability of disease.

**Fig. 3.**
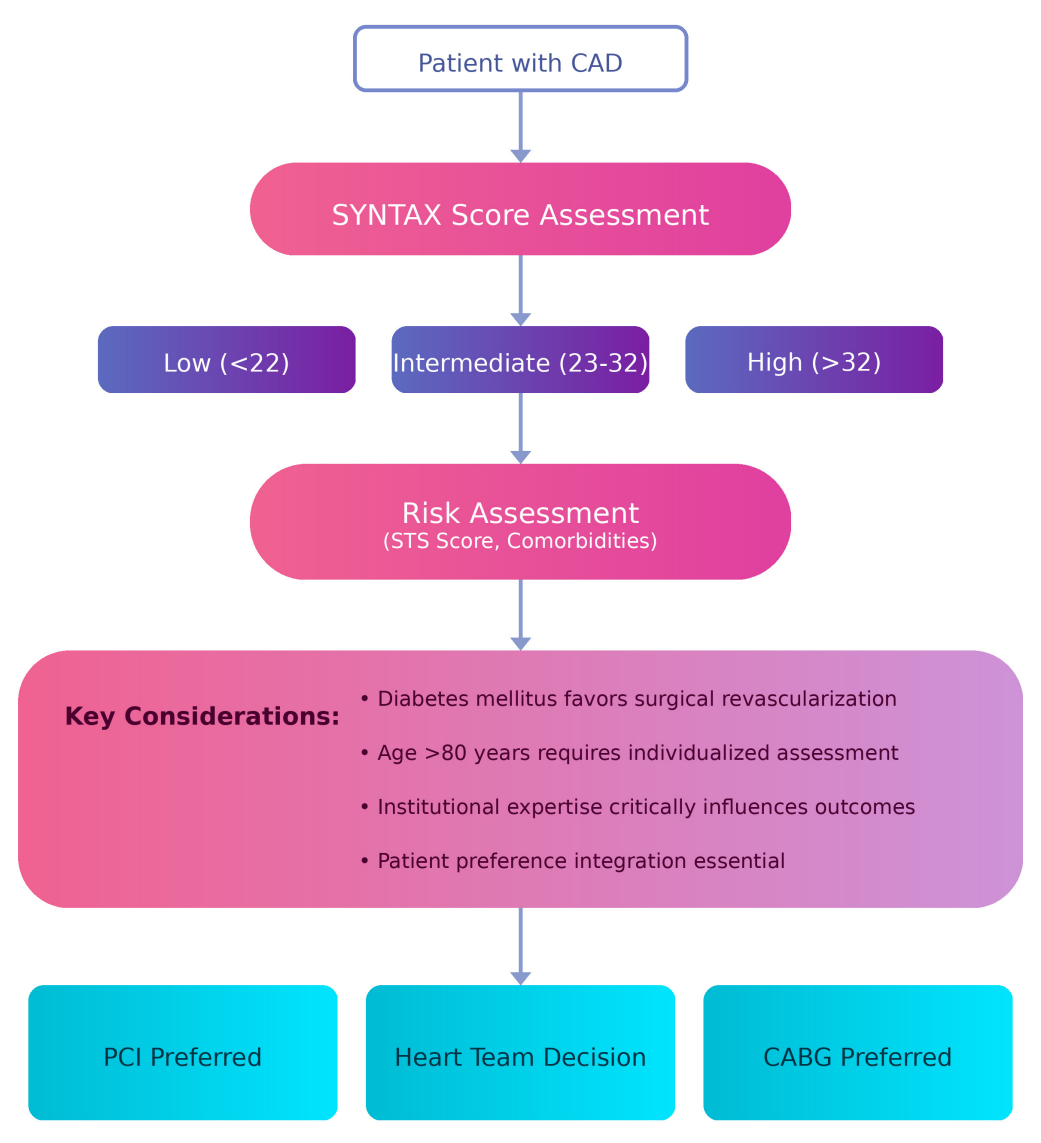
**Evidence-based clinical decision algorithm for contemporary myocardial revascularization incorporating anatomical complexity, patient factors, and institutional capabilities**. STS, Society of Thoracic Surgeons; CAD, Coronary Artery Disease. Figure created with Biorender.

Limitations include beam-hardening artifacts in heavily calcified vessels, reduced image quality with irregular heart rhythms, and contraindications in patients with contrast allergy, renal dysfunction, or severe obesity. Patient-specific determinants are increasingly guiding treatment selection, and systematic consideration should encompass frailty, nutritional status, cognitive function, and genetic evaluations for diseases that affect procedural risk [62, 63, 64, 65]. Institutional determinants such as operator experience, resource availability, and quality improvement programs also play an important role in optimal treatment selection (Fig. 3) [66, 67, 68].

### 5.2 Quality Metrics and Continuous Improvement

#### Contemporary Quality Measurement

Quality improvement in myocardial revascularization demands a holistic approach that encompasses procedural success, patient-oriented outcomes, and the duration of efficacy [69]. Modern quality metrics increasingly favor composite endpoints that quantify the overall patient experience rather than isolated procedural metrics.

Procedural quality measures encompass technical success and high complication rates, while clinical outcome measures focus on adverse event rates such as mortality, myocardial infarction, stroke, and acute kidney injury [70, 71]. Patient-centered measures assess improvements in functional status, health-related quality of life, and patient-satisfaction scores [72].

System-level measures evaluate the effectiveness of healthcare delivery by optimizing length of stay, reducing readmission rates, and optimizing resource utilization [73]. Long-term quality measures focus on survival benefit, freedom from repeat revascularization, and persistent functional improvement [74].

## 6. Persistent Challenges and Future Directions

### 6.1 Healthcare Disparities: Addressing Inequities

#### Geographic and Socioeconomic Barriers

Rural–urban differences persist, with rural hospital patients exhibiting lower rates of utilization of therapeutic interventions and higher mortality for cardiovascular disease [3]. Geographic differences need to be addressed through systematic approaches, including wide strategies on telemedicine platforms, regional networks of care, rapid transport systems with uniform procedures, and wide provider education programs [75, 76, 77, 78, 79].

Racial differences in the use of coronary artery bypass grafting and in mortality persist with wide regional variation, and gender differences in detection, referral, and treatment also persist to influence outcomes [80].

### 6.2 Late Complications: Evolving Management Strategies

#### 6.2.1 In-Stent Restenosis: Contemporary Approaches

Despite advances in current drug-eluting stents, failure mechanisms remain dynamic. Neoatherosclerosis has become an increasingly important consideration, in addition to classic neointimal proliferation [81, 82]. Modern management requires comprehensive prevention strategies, including optimal initial techniques, systematic follow-up, and evidence-based treatment of established restenosis [83, 84, 85, 86, 87].

#### 6.2.2 Graft Failure: Understanding and Prevention

Graft failure following coronary artery bypass grafting occurs in 10–50% of cases, depending on the conduit used. Early graft failure is usually clinically silent but is associated with increased mortality [88]. Prevention is a concerted effort that involves optimal surgical technique, aggressive medical management, routine surveillance measures, and early intervention for early dysfunction [89, 90, 91, 92].

### 6.3 Future Technological Horizons

#### 6.3.1 Artificial Intelligence Integration (2025–2035)

**Fig. 4.**
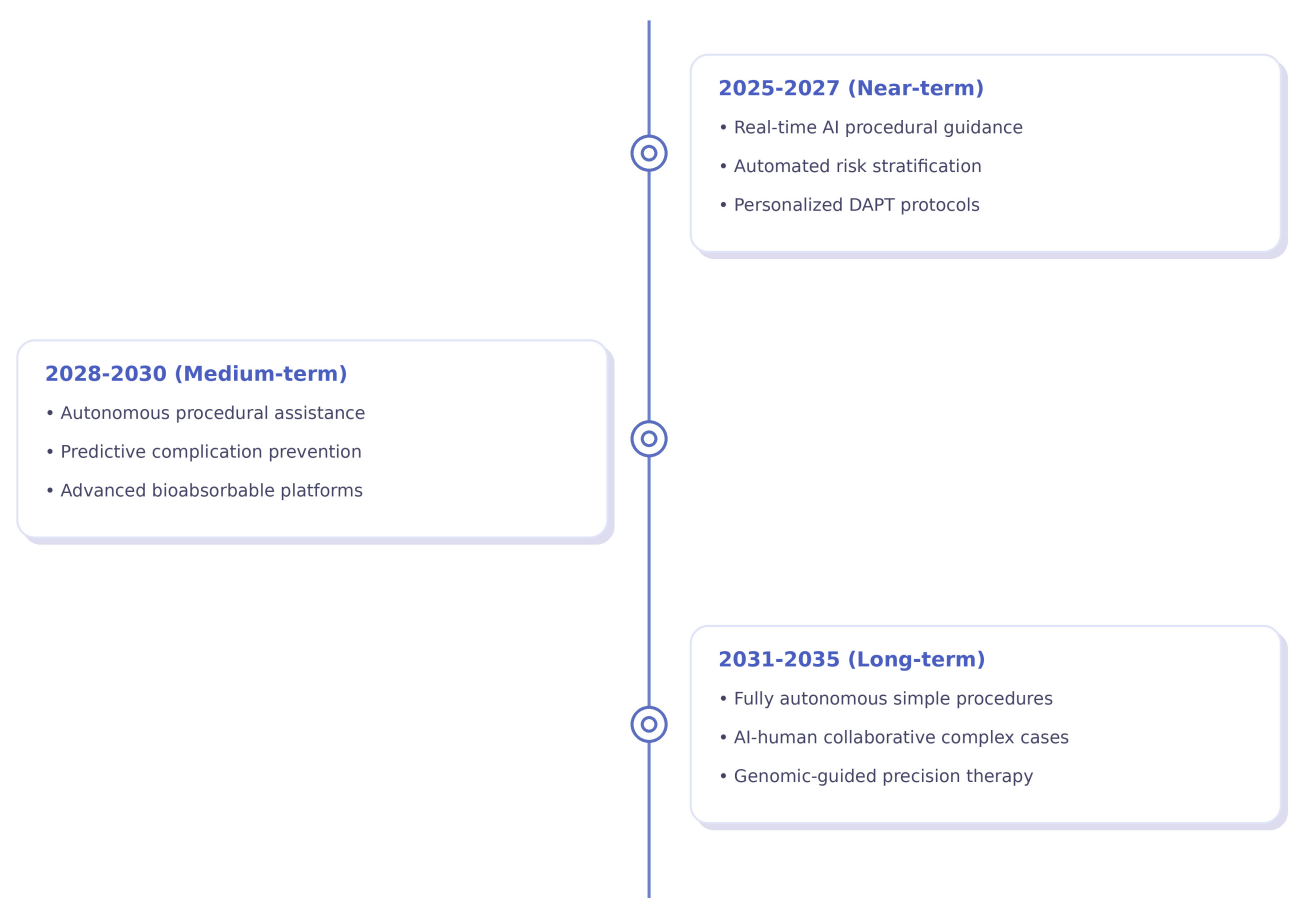
**Projected timeline for artificial intelligence and precision medicine integration in cardiovascular care, with implementation phases based on current development trajectories**. DAPT, Dual Anti-Platelet Therapy. Figure created with Biorender.

Over the next decade, AI will be increasingly integrated into all aspects of revascularization. Short-term innovations (2025–2027) are expected to include real-time procedural guidance systems, automated risk stratification, and individualized medication protocols. Meanwhile, medium-term advancements (2028–2030) are expected to include autonomous procedural support and complication prevention using predictive analytics. Longer-term applications (2031–2035) may involve totally autonomous, uncomplicated procedures and AI–human cooperative management of complex interventions (Fig. 4) [93, 94, 95, 96, 97].

#### 6.3.2 Advanced Materials and Precision Medicine

Advances in bioabsorbable scaffold technology are moving toward clinically relevant systems that provide temporary support to promote vessel healing. Whole-genome assessment will facilitate the prediction of procedural success, guide optimal device selection, and tailor medical therapy. Meanwhile, integrating clinical, laboratory, imaging, and genomic data with machine learning will transform patient selection and outcome prediction.

## 7. Clinical Recommendations and Implementation

### 7.1 Immediate Practice Implications (2025–2026)

#### 7.1.1 Evidence-Based Recommendations

∙ Adopt a comprehensive patient assessment that incorporates frailty, nutritional status, and cognitive evaluation alongside traditional cardiac risk factors.

∙ Implement systematic quality improvement programs that prioritize patient-centered outcomes.

∙ Develop institutional heart team protocols for complex cases that require multidisciplinary decision-making.

∙ Prioritize reducing healthcare disparities through systematic evaluation and targeted intervention programs.

∙ Invest in AI-supported decision-making tools as these applications become clinically validated.

#### 7.1.2 Technology Adoption Strategy

Early adoption should be directed toward technologies with strong evidence bases and established clinical benefits [98]. New-generation drug-eluting stents, with extensive safety datasets, should be the foundation for intervention programs, with the newer technologies added only after rigorous evaluation [4]. Systematic application of intracoronary imaging in complex patients provides near-term benefits while developing expertise for future use [99].

Physiological evaluation with fractional flow reserve is expected to become standard for intermediate lesions, with demonstrated clinical utility and cost-benefit [100]. Robotic surgical programs should be selectively developed in hospitals with sufficient volume and infrastructure support [101].

### 7.2 Strategic Healthcare System Planning

Modern healthcare systems need to implement a comprehensive infrastructure to support integrated care delivery models across the entire revascularization continuum and to future-proof for anticipated emerging technologies [102, 103, 104, 105, 106, 107]. Technology expenditures should focus on AI-integration capabilities, comprehensive data analytics, and unfettered information sharing [108]. Training programs need to ensure proficiency with evolving techniques and develop adaptability to new technologies [109].

Quality improvement models should include robust outcome tracking in addition to traditional procedural measures, including patient-reported outcomes and long-term effectiveness [110]. Care coordination infrastructure must enable unobstructed patient movement along the continuum, from acute care to long-term follow-up [111].

## 8. Conclusions

Myocardial revascularization practice in 2025 reflects dramatic progress in reducing the global burden of coronary artery disease. Current drug-eluting stents are delivered with unparalleled safety and effectiveness, and surgical methods continue to achieve optimal outcomes for complicated cases, particularly with the advent of minimally invasive techniques. The advent of artificial intelligence, robotic surgery, and precision medicine has the potential to transcend current limitations while expanding treatment options for high-risk patient populations.

Major challenges remain, such as health disparities that limit access to optimal therapy and late complications, including restenosis and graft failure. The growing complexity of the patient population threatens classical risk–benefit considerations and necessitates novel strategies for patient selection and care delivery.

The future lies in harmonization, not competition, between new technology and established practice. Success depends on keeping patient-centered outcomes at the forefront and accepting innovations that demonstrably enhance the quality and accessibility of care. The heart team model has evolved from simple consultation to obligatory collaboration, enabling optimal strategy selection across the entire revascularization continuum.

The future of myocardial revascularization will be guided by our ability to capitalize on technological innovation without compromising the fundamental principles of evidence-based practice and compassionate patient care. Achieving this vision will require continued interdisciplinary collaboration, a sustained commitment to reducing healthcare disparities, and a focus on maximizing outcomes for all people affected by coronary artery disease.
